# The HMW1C-Like Glycosyltransferases—An Enzyme Family with a Sweet Tooth for Simple Sugars

**DOI:** 10.1371/journal.ppat.1003977

**Published:** 2014-04-10

**Authors:** Jessica R. McCann, Joseph W. St. Geme

**Affiliations:** 1 The Department of Pediatrics, Duke University Medical Center, Durham, North Carolina, United States of America; 2 The Department of Pediatrics, The Children's Hospital of Philadelphia and the Perelman School of Medicine at the University of Pennsylvania, Philadelphia, Pennsylvania, United States of America; The University of North Carolina at Chapel Hill, United States of America

## The HMW1/HMW2 Two-Partner Secretion Systems Have a Third Partner

The HMW1 and HMW2 adhesins of nontypeable *Haemophilus influenzae* are high-molecular weight proteins that are secreted by the two-partner secretion (TPS) pathway, also known as the Type Vb secretion pathway [1], [2]. TPS systems typically consist of a large extracellular protein called a TpsA protein (encoded by a *tpsA* gene) and a cognate outer membrane pore-forming translocator protein called a TpsB protein (encoded by a *tpsB* gene). HMW1 and HMW2 are TpsA proteins and are encoded by *hmw1A* and *hmw2A*, respectively, and HMW1B and HMW2B are the cognate TpsB proteins and are encoded by *hmw1B* and *hmw2B*, respectively [3], [4]. The *hmw1A-hmw1B* and *hmw2A-hmw2B* gene clusters have a similar configuration and are located in physically separate regions of the *H. influenzae* chromosome.

A distinctive feature of the HMW1 and HMW2 systems is the presence of a third protein, called HMW1C in the HMW1 system and HMW2C in the HMW2 system. HMW1C and HMW2C are highly homologous glycosyltransferases [5], [6] that are responsible for adding sugar moieties to HMW1 and HMW2 and are encoded by the *hmw1C* and *hmw2C* genes, located downstream of *hmw1B* and *hmw2B*, respectively. Since the HMW1 and HMW2 systems have similar properties [7], in this review we will confine our discussion to the HMW1 system.

**Figure 1:**
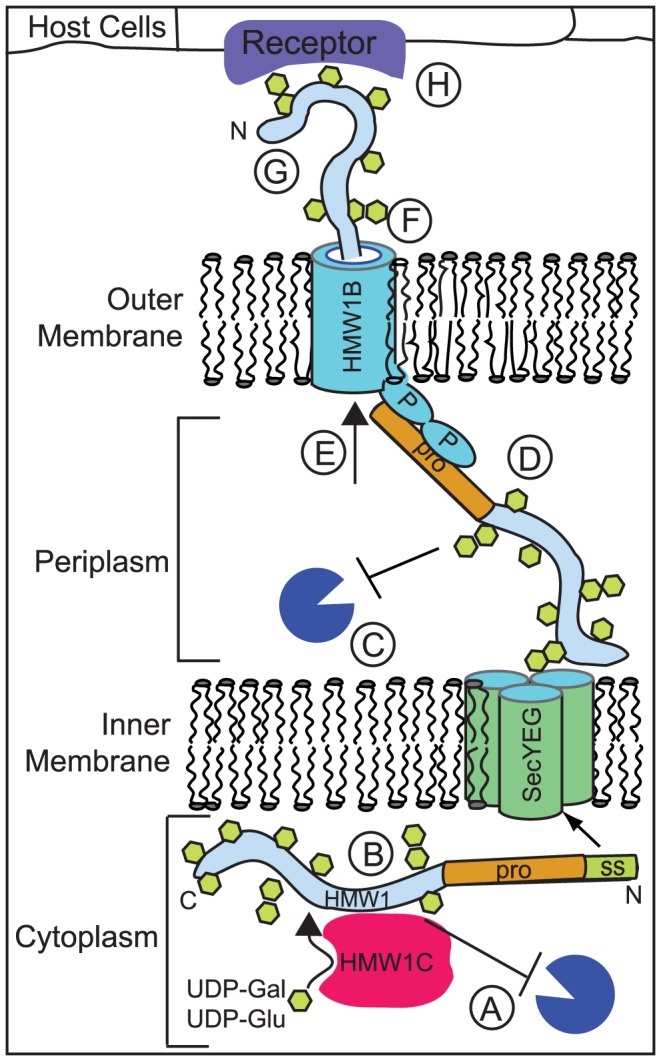
Glycosylation by HMW1C may play several roles in promoting HMW1 stability, export, folding, and function. HMW1C has the potential to contribute to several different processes that occur during HMW1 synthesis and transit across the inner and outer membranes. First of all, HMW1C glycosylates the HMW1 adhesin in the cytoplasm and is likely to be involved in the stability of the HMW1 adhesin during or after its synthesis. Glycosylation may contribute to stability of HMW1 in the (**A**) cytoplasm or (**C**) periplasm [5], [7]. Alternatively, the HMW1C protein may improve stability of HMW1 by acting as a (**B**) chaperone prior to secretion of the adhesin. It is unlikely that the activity of HMW1C is required for export of the adhesin across either the inner or outer membrane, as fully processed HMW1 is found in the supernatant in the absence of HMW1C [5]. It is unclear whether glycosylation influences interaction of HMW1 with the (**D**) HMW1B periplasmic domain prior to transit, (**E**) the HMW1B pore during transit, or (**F**) the docking region of HMW1B upon surface tethering [5], [11]. It is also unclear whether glycosylation participates in (**G**) protein folding upon export. Evidence from the nonglycosylated *Bordetella* prototypic, two-partner, secreted adhesin FHA indicates that this adhesin remains unfolded in the cytoplasm and folds very rapidly upon export via its TpsB secretion pore [30]. One hypothesis is that the energy generated by this rapid folding is at least part of what drives export of TpsA proteins across the outer membrane [2]. Finally, glycosylation of HMW1 may be required for (**H**) adherence to host cells or host interaction in a particular niche.

The HMW1 adhesin is presented on the bacterial surface via a multistep process that requires HMW1C-mediated glycosylation (reviewed in [8]). As shown schematically in Figure 1, HMW1 is synthesized and glycosylated in the cytoplasm and is directed to the Sec translocase in the inner membrane via an extended N-terminal signal sequence [9]. The signal sequence is cleaved by signal peptidase I, and nascent HMW1 is then directed to its cognate HMW1B β-barrel pore in the outer membrane [9]. The initial interaction between HMW1 and HMW1B occurs via the N-terminal TPS secretion domain in the HMW1 pro-piece and the periplasmic domain in HMW1B [10]. The HMW1 pro-piece spans amino acids 69–441 and is cleaved during or following secretion through the HMW1B pore [9]. HMW1 is ultimately tethered to the bacterial surface via a noncovalent interaction that requires the C-terminal 20 amino acids of the protein and is dependent upon disulfide bond formation between two conserved cysteine residues in this region (cysteines 1518 and 1528). Immunolabeling studies have demonstrated that the immediate C terminus of HMW1 is inaccessible to surface labeling, suggesting that it remains in the periplasm or is buried in the HMW1B pore [5], [11]. Elimination of HMW1C results in degradation of HMW1 in bacterial lysates, indicating that glycosylation is required for HMW1 stability. Any remaining nonglycosylated HMW1 is released into the culture supernatant, indicating that glycosylation is also required for HMW1 tethering to the bacterial surface [5].

Manual analysis of mass spectra of HMW1 was required to recognize that glycan structures are present at asparagine residues in conserved NXS/T motifs, reflecting the fact that the modifying carbohydrates are mono-hexose or di-hexose groups rather than complex polysaccharides [12], [13]. There are at least 31 residues that are modified with glucose, galactose, glucose-glucose, or glucose-galactose residues in the mature surface-localized HMW1 protein [12], [13]. Based on biochemical analysis and examination of the crystal structure of the HMW1 pro-piece, the pro-piece is nonglycosylated, perhaps because glycosylation would interfere with cleavage of this fragment, which occurs by an undefined mechanism (Figure 1) [9], [12], [14].

## HMW1C Is the Prototype Member of a New Subfamily of Glycosyltransferases

Protein glycosylation occurs in all kingdoms of life and is thought to influence protein folding, stability, and function [15], [16]. Some bacteria produce complex *O*-linked or *N*-linked glycosyltransferase systems. These systems have been studied in pathogenic bacteria and glycosylate proteins that are typically surface exposed, suggesting a role for glycosylation in bacteria–host interactions [17]. However, none of the previously studied bacterial glycosyltransferase pathways operates like HMW1C, which is capable by itself of forming both *N*-linked carbohydrate bonds to the HMW1 polypeptide and *O*-glycosidic bonds between hexose sugars [12], [18], [19].

Based on homology analysis and molecular modeling, HMW1C belongs to the GT41 family of glycosyltransferases, a family that otherwise contains *O-*GlcNAc transferases. HMW1C consists of three discrete domains, including an α-helical AAD domain at the N terminus and two Rossman-like domains that create a GT-B fold at the C terminus. Interestingly, the AAD fold in HMW1C differs from the so-called tetratricopeptide repeats (TPR) fold that is characteristic of the GT41 family, and the contacts between the AAD domain and the GT-B domain in HMW1C create a unique groove that is absent in other known members of the GT41 family. Thus, the HMW1C protein represents a novel glycosyltransferase subfamily [13].

Among the best-described bacterial *N*-linked glycosyltransferase systems is the Pgl system in *Campylobacter jejuni* [20]–[22]. While both the Pgl system and HMW1C affix sugars to their target proteins at asparagines, the similarities end there. First, the Pgl system consists of at least ten proteins encoded by a gene cluster [23], rather than a single protein like HMW1C. Second, the Pgl enzymes are active in the periplasm, while HMW1C acts in the cytoplasm [5]. Third, the Pgl enzymes add a heptasaccharide that is most likely formed in the cytoplasm on a lipid carrier that is then flipped into the periplasm. In contrast, HMW1C adds single UDP-linked sugars to HMW1 without the contribution of a lipid carrier [12].

## The HMW1C-Like Glycosyltransferases Segregate into Two Subsets

**Figure 2:**
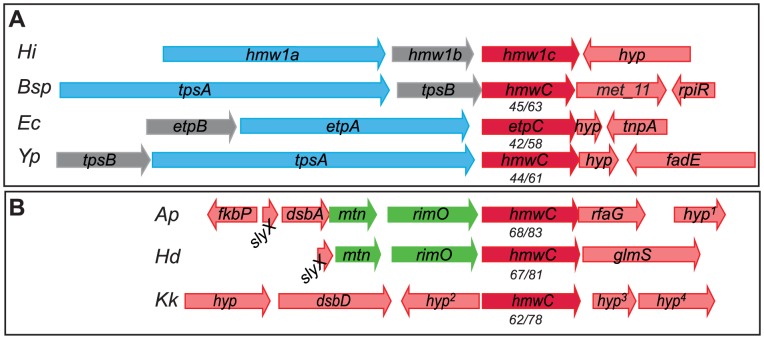
HMW1C-like proteins in two categories: Those encoded by loci that contain obvious substrate genes and those encoded by isolated genes without adjacent substrate genes. Numbers below *hmw1C*-like genes represent translated protein sequence percent identity/similarity when compared to *H. influenzae* HMW1C. (**A**) HMW1C-like enzymes encoded in apparent TPS systems. (**B**) HMW1C-like enzymes encoded in loci without obvious surface protein targets for glycosylation. Abbreviations: *Hi*, *H. influenzae* 86-028NP; *Bsp*, *Burkholderia* species GCE1003; *Ec*, Enterotoxigenic *E. coli* H10407; *Yp*, *Y. pseudotuberculosis* YPIII; *Ap*, *Actinobacillus pleuropneumoniae* L20; *Hd*, *H. ducreyi* HD35000; *Kk*, *K. kingae* 269–492; *hyp*, hypothetical with no conserved domains; *hyp* ^1^, predicted lipoprotein; *hyp* ^2^, predicted UDP-glcNAc carboxyvinyltransferase; *hyp* ^3^, predicted 2 C-methyl-D erythritol-4-phosphate cytidyltransferase; *hyp* ^4^, predicted deoxyguanosinetriphosphate triphosphohydrolase.

Based on homology analysis of predicted amino acid sequences, HMW1C-like proteins are prevalent among bacteria in the Pastuereallaceae, Enterobacteriaceae, Neisseriaceae, and Burkholderiaceae families and appear to segregate into two categories [24]. The first category contains enzymes encoded by genes adjacent to predicted *tpsA* and *tpsB* genes in apparent TPS systems (Figure 2A). Examples of HMW1C-like proteins that fall into this category are EtpC in enterotoxigenic *Escherichia coli* (ETEC) [25], RscC in *Yersinia enterocolitica* [26], and predicted HMW1C-like glycosyltransferases in *Y. pestis*, *Y. pseudotuberculosis*, and *Burkholderia* spp. Among these proteins, only EtpC has been demonstrated to possess glycosyltransferase activity, adding sugar residues to the target EtpA adhesin [25]. In fact, glycosylation of EtpA appears to affect adhesin interaction with host cells, as nonglycosylated EtpA is less adherent to Caco-2 intestinal epithelial cells but hyperadherent to HCT-8 intestinal cells when compared to glycosylated EtpA [25]. By analogy to the HMW1 system and the Etp system, we hypothesize that the HMW1C-like enzymes in this category modify the co-produced TpsA protein.

The second category of HMW1C-like proteins contains enzymes that are encoded by genes adjacent to genes encoding ribosomal proteins and other transferase enzymes involved in carbohydrate modification. Members of this category have no obvious predicted protein target for glycosylation and include the crystallized *Actinobacillus pleuropneumoniae* HMW1C-like protein (ApHMW1C) and predicted HMW1C-like proteins in *H. ducreyi* and *Kingella kingae* (Figure 2B). Of the proteins in this category, only ApHMW1C has been characterized and is known to possess glycotransferase activity [18], [19], [27]. Recent work has demonstrated that the ApHMW1C glycosyltransferase preferentially decorates predicted autotransporter proteins as well as other outer membrane proteins when expressed in vivo and in *E. coli* [27], suggesting that enzymes in this category may generally have a preference for glycosylation of outer membrane proteins. It is interesting to note that the HMW1C-like glycosyltransferases in this second category are more homologous to the prototype HMW1C protein than are the HMW1C-like proteins encoded by TPS loci.

## HMW1C-Like Enzymes Have a Well-Defined Structure Involved in Binding and Transferring UDP-Hexoses

The crystal structure of ApHMW1C provides some clues as to how HMW1C-like glycosyltransferases are able to decorate asparagines. When overlaid using structural prediction algorithms, HMW1C and ApHMW1C are nearly identical, differing only by a disordered 30-amino acid N-terminal tail that is present in HMW1C and absent in ApHMW1C [19]. Given this level of identity, predictions about structure-function relationships in ApHMW1C are likely to apply to *H. influenzae* HMW1C and potentially other HMW1C-like proteins. Consistent with this conclusion, mutation of amino acids in HMW1C corresponding to amino acids that line the likely UDP-hexose binding pocket in ApHMW1C eliminates the ability of HMW1C to glycosylate HMW1 or to produce functional HMW1 when expressed in whole bacteria [19]. Examination of the ApHMW1C structure indicates a funnel-shaped groove immediately adjacent to the predicted UDP-hexose binding pocket. When key residues within this groove are mutated in HMW1C, glycosylation of HMW1 is eliminated, indicating that the groove plays an important role in acceptor protein modification [19]. A complete understanding of the specificity and limitations of the HMW1C-like enzyme family may help in an industrial setting, as a single enzyme able to catalyze the first step of *N*-linked protein glycosylation without a lipid carrier has potential to become a workhorse for in vitro protein production [28].

## What Do We Still Want to Learn About HMW1C-Like Glycosyltransferases?

Despite the relative simplicity of HMW1C-mediated glycosylation, there is still much to learn about how the HMW1C prototype operates and even more to learn about the function and targets of HMW1C homologs in other medically relevant bacteria. First, we do not understand how HMW1C recognizes its target and chooses specific glycosylation sites, as only a subset of NXS/T motifs in HMW1 are decorated based on mass spectrometry analysis. A better understanding of HMW1C target recognition may assist in development of in vitro glycosylation systems for protein manufacture and also increase understanding of protein–protein interactions in bacteria. Second, while it is clear that HMW1C interacts directly with HMW1 [5], the number of HMW1C molecules that bind to HMW1 at any given time and the duration of this interaction in the cytoplasm are still unknown. This information may help to clarify whether HMW1C has both glycosyltransferase activity and chaperone activity to stave off degradation of HMW1 (Figure 1). Third, the glycosylation targets in bacteria that have no co-transcribed *tpsA* gene alongside the gene that codes for the HMW1C-like enzyme remain to be determined, although progress has been made in identifying potential targets of ApHMW1C [27]. Knowledge of these targets will help to clarify whether there are consensus glycosylation target sequences among HMW1C-like enzymes and whether the cellular location of glycosylation is conserved. Fourth, while it is clear that glycosylation is required for HMW1 tethering to the bacterial surface, the mechanism involved is unknown. Finally, it will also be important to elucidate how HMW1C-mediated glycosylation of target proteins affects bacterial pathogenesis, expanding the limited literature on the role that HMW1C-mediated glycosylation may play in bacteria–host interactions [19], [25], [26], [29].

The HMW1C-like glycosyltransferases have novel features, and advances in our understanding of this interesting group of enzymes will likely have important implications for the field of glycobiology and the field of bacterial pathogenesis.
